# Clinical and pathological report of an unusual anterior chamber lesion: A case report

**DOI:** 10.1186/1757-1626-1-145

**Published:** 2008-09-08

**Authors:** Dany M Najjar, Sagun J Pendse, Julianne C Lin, Pecos T Olurin

**Affiliations:** 1 Department of Ophthalmology, Temple University Hospital, Philadelphia, PA, USA; 2 Department of Ophthalmology, Wilmington Hospital, Wilmington, DE, USA

## Abstract

To describe an unusual anterior chamber lesion found on routine eye examination of a 71 year-old Hispanic gentleman who presented for cataract evaluation. The lesion was biopsied at the time of cataract surgery and its pathology presented.

A 71 year-old Hispanic gentleman presented for routine cataract evaluation. We found an unusual lesion in the anterior chamber of the right eye. The patient underwent uneventful phacoemulsification surgery. The lesion was biopsied at the time of cataract surgery and sent for pathology. Clinical photos and its pathology are presented in this article.

Despite biopsy and several ancillary testings, the nature of this lesion remains unknown. Only long-term follow-up of the left eye might reveal clues as to the origins of this unusual lesion.

## Introduction

We describe an unusual anterior chamber lesion found on routine eye examination of a 71 year-old gentleman who presented for cataract evaluation. The lesion was biopsied at the time of cataract surgery and its pathology presented.

## Case presentation

**Figure 1:**
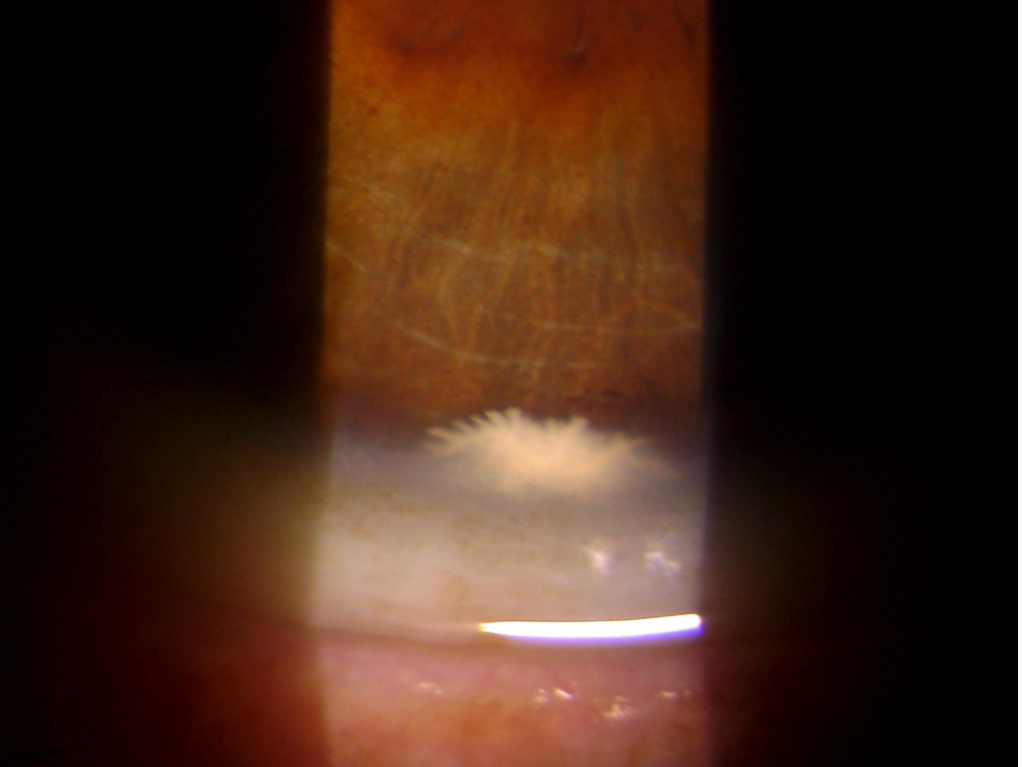
Anterior chamber lesion of the right eye, found on routine eye examination of a 71 year-old patient.

**Figure 2:**
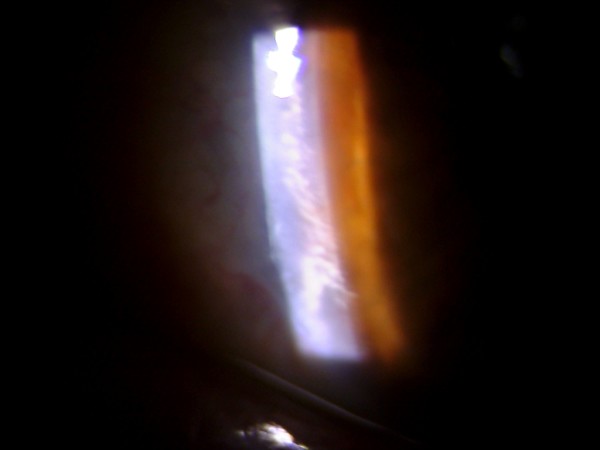
The left eye of the same patient shows multiple small whitish crystalline projections at the nasal limbus.

A 71 year-old gentleman presented to our clinic for cataract evaluation. His past medical history was negative except for well-controlled type II diabetes. On examination, his vision was 20/70 in the right eye and 20/50 in the left. His intraocular pressures were 15 mm Hg in both eyes. Slit lamp examination revealed a quiet conjunctiva in both eyes, as well as nuclear sclerosis cataracts consistent with his visual acuity. Posterior segment examination did not show any diabetic retinopathy. In addition, we noticed an unusual lesion in the anterior chamber of the right eye. The lesion was pearly white, solid looking and had multiple finger-like projections (Figure 1). It was located just behind the posterior corneal stroma inferiorly in the anterior chamber. Gonioscopy revealed an open angle with normal angle structures, and no extension of the mass into the angle. The cornea was completely normal otherwise. The anterior chamber was quiet. Examination of the left eye was remarkable for multiple small whitish crystalline projections at the nasal limbus (Figure 2). The patient denied any history of ocular trauma, ocular infections or drug abuse.

**Figure 3:**
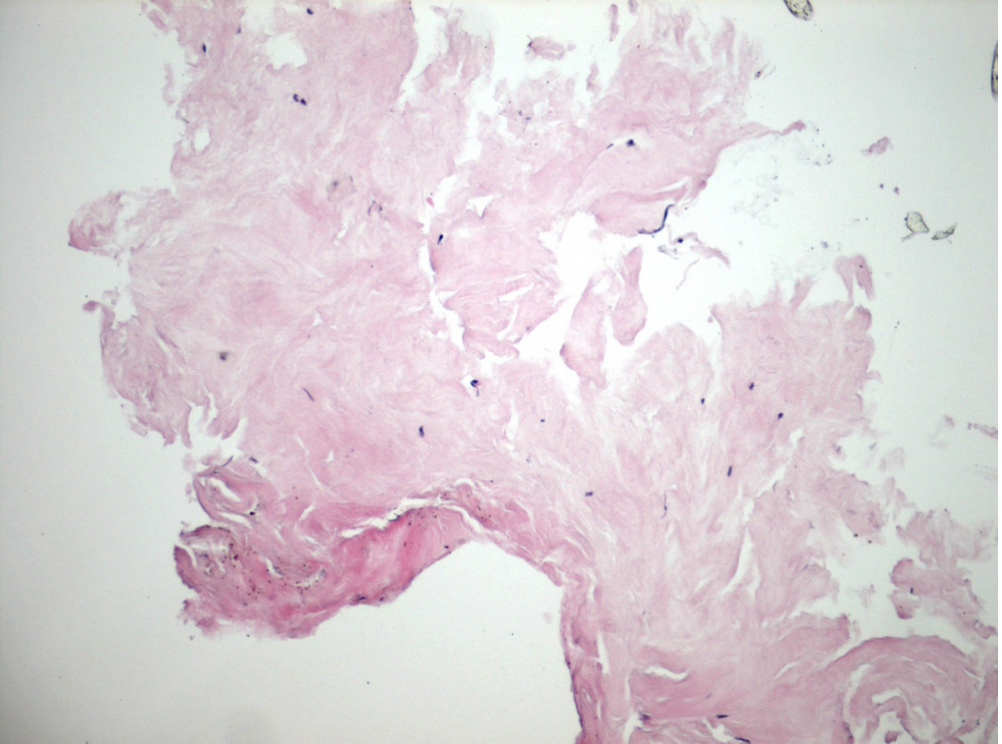
Pathology of the described lesion revealed paucicellular eosinophilic material consistent with connective tissue.

Due to the unusual nature of the lesion, an oncology consultation was requested. A plain radiographic film of the orbit was obtained and did not reveal any calcification. Blood levels of calcium and magnesium were normal. The patient underwent an uneventful phacoemulsification surgery of the right eye with excisional biopsy of the lesion at the time of cataract surgery. The lesion was strongly adherent to the posterior corneal stroma and was rubbery in nature, requiring dissection into the cornea. It was removed in small pieces, and sent for pathological identification. Pathology of the mass revealed a paucicellular eosinophilic material, mildly PAS positive, and consistent with connective tissue. Stains for amyloid were negative (Figure 3).

## Conclusion

A review of the literature using PubMed has revealed previously reported cases of anterior segment epibulbar choristomas containing brain tissue arising from the cornea [1,2]. However, in our case, pathology did not reveal any abnormal tissue, and it is unknown whether the patient was born with this lesion or whether it was acquired. Other entities to consider in the differential diagnosis include lesions arising from the iris or ciliary body and extending into the cornea and anterior chamber [3]. However this was not the case as it was ruled out by gonioscopy. Stone et al reported a case of metaplastic squamous epithelial downgrowth occurring after clear corneal cataract surgery [4]. Our patient had not undergone any ocular surgeries prior to his initial visit, and the eye was quiet without any signs of inflammation.

Questions as to the etiology of this lesion remain unanswered. Did this lesion originate in the posterior cornea and then protrude in the anterior chamber, or did it originate in the anterior chamber and got stuck to the posterior cornea? Could it represent some form of hamartomatous proliferation of the posterior stroma, or was it acquired? Only long-term follow-up of the left eye might reveal some clues as to the origins of this unusual anterior chamber lesion.

## Competing interests

The authors declare that they have no competing interests.

## Authors' contributions

SP examined the patient and took pictures of the lesion. PO examined the patient and recommended ancillary testing and appropriate consultations. DN and JL examined the patient and recommended appropriate testing and consultations. They also performed the cataract surgery and biopsy of the lesion. The pathology specimen was sent to Wills Eye Institute to be examined by Ralph Eagle, M.D. DN compiled all the data and was a major contributor in writing the manuscript. All authors read and approved the final manuscript.

## Consent

Written informed consent was obtained from the patient for publication of this case report and accompanying images. A copy of the written consent is available for review by the Editor-in-Chief of this journal.
